# Decolourization of Diazo Evans Blue by Two Strains of *Pseudomonas fluorescens* Isolated from Different Wastewater Treatment Plants

**DOI:** 10.1007/s11270-012-1276-4

**Published:** 2012-07-31

**Authors:** Ewa Zabłocka-Godlewska, Wioletta Przystaś, Elżbieta Grabińska-Sota

**Affiliations:** Environmental Biotechnology Department, Silesian University of Technology, Akademicka 2A, 44-100 Gliwice, Poland

**Keywords:** *Pseudomonas*, Azo dye, Decolourization, Zootoxicity, Phytotoxicity

## Abstract

The use of azo dyes is popular in different branches of industry. Discharge of colourants to surface water cause harmful environmental effects. The aim of the present study was evaluation of effectiveness of diazo Evans blue decolourization by two *Pseudomonas* strains and estimation of process byproducts toxicity. In static conditions, both tested strains removed more than 85 % of dye after 48 h and completely decolorized samples after 120 h. Agitation had negative impact on Evans blue removal (less than 70 % of dye removed after 120 h). Ecotoxicological effects were different for both studied strains beside comparable decolourization effectiveness. Increase of zootoxicity was noticed for strain Sz6 and decrease from IV to III class was noticed for strain SDz3. Optimization of process conditions for the most promising strain SDz3 should be deeply examined.

## Introduction

Dyes applied in industry are designed to resist light, water, oxidizing agents, and microbial attack. They are used for colouring textiles, leather, paper, food, cosmetics, medical treatment and analysis (Swamy and Ramsay 1999; Padamavathy et al. 2003; Somasiri et al. 2006). Around 10,000 different dyes are commercially available. Imperfection of textile colouration processes cause even 10–15 % losses of applied dyes and environmental pollution. In addition to their visual effect, they increase chemical oxygen demand and reduce light penetration in surface water. Many of them are known as toxic, mutagenic and carcinogenic substances (Gill et al. 2001).

Colour reduction may be achieved by physicochemical as well as biological processes. The conventional physicochemical methods for removal of dyes include flocculation, flotation, precipitation, oxidation, reduction, ozonation, coagulation, membrane separation and adsorption. These methods of dye decolourization/degradation cause some unresolved problems. They are financially and often also methodologically demanding, time-consuming and sometimes mostly not very effective (Hu 2001; Eichlerova et al. 2006). Despite the existence of a variety of physical and chemical methods a promising technology appears to be biodegradation. Microbial decolourization is an environmentally friendly and cost-competitive alternative (Verma and Madamwar 2003).

It is reported that some anaerobic and microaerophilic bacteria decolorize azo dyes effectively (Hu 2001; Eichlerova et al. 2006; Stolz 2001). The first step is cleavage of azo bond by azoreductases activity and formation of colorless aromatic amines that are carcinogenic and mostly more toxic than the starting compounds (Hu 2001; Eichlerova et al. 2006; Stolz 2001; Verma and Madamwar 2003; Van der Zee and Villaverde 2005). Further degradation of aromatic amines generally requires aerobic conditions (Van der Zee and Villaverde 2005). Azoreductases have been identified in several bacteria, such as *Klebsiella pneumoniae* RS-13, *Pseudomonas luteola, Xenophilus azovorans* KF46 (Pointing and Vrijmoed 2000), *Rhodococcus* (Fu and Viraraghavan 2001), *Shigella dysenteriae* Type I, *Escherichia coli* and *Clostridium perfringens* (Moreira et al. 2000; Bin et al. 2004).

Most reported research is related to azo dyes decolourization. Information on the toxicity of the biotransformation products is however limited. In the present study we focused our attention on effectiveness of dyes removal by *Pseudomonas* bacteria as well as toxicological effects of decolourization products on water organisms.

## Materials and Methods

### Isolation of Bacterial Strains

Bacterial strains were isolated from two different wastewater treatment plants (Katowice, Swibie, south Poland). Daily flow of wastewater treatment in Swibie was 250 m^3^/day (sewage farm) and in Katowice 43,000 m^3^/day. Samples of sewage were taken from inlet to wastewater treatment plant in Katowice and from biological ponds in Swibie. Isolation was done on Nutrient Agar (BTL) with 0.1 g l^−1^ of brilliant green. Two strains (Sz6 originate from Swibie and SDz3 from Katowice) with highest effectiveness of decolourization in isolation phase were chosen to the experiment. They were classified by the API 20NE test (Biomerieux) as *Pseudomonas fluorescens*.

### Decolourization Potential

Experiment was performed on Kimura medium (glucose 20 g l^−1^, peptone 5 g l^−1^, yeast extract 2 g l^−1^, MgSO_4_ 0.5 g l^−1^, KH_2_PO_4_ 1 g l^−1^, pH 6.8). Medium was inoculated with bacterial suspension in physiological salt with optical density 5 in McFarland scale (∼15 × 10^8^ cfu ml^−1^). Five different dyes were chosen for decolourization potential test: fluorone (Bengal rose, erythrosine), triphenylmethane (brilliant green, crystal violet), azo dye (Evans blue). Dyes were used in two concentrations: 0.05 and 0.1 g l^−1^. Filter-sterilized colourants were added to tubes containing 10 ml of 48-h-old cultures in stationary growth phase. Such long time of bacteria strains incubation was necessary for obtaining required concentration of biomass (∼15 × 10^8^ cfu ml^−1^). All samples were done in triplicate. After 6 days of incubation in 26 °C, absorbance was measured spectrophotometrically on UV–Vis Hitachi U-1900. Maximal absorbance was noticed at 527 nm for erythrosine, 548 nm for Bengal rose, 590 nm for crystal violet, 624 nm for brilliant green and 606 nm for Evans blue. Percentage dye removal was calculated according to Formula 1.

1 $$ R = \left( {{{{\left( {C - S} \right)}} \left/ {C} \right.}} \right) \times 100\% $$

*R*: Dye removal [%]
*C*: Concentration of dye in a control sample [mg l^−1^]
*S*: Residue concentration of dye in samples with bacteria biomass [mg l^−1^]

### Decolourization of Evans Blue

Main experiment was preceded by test concerning the influence of Evans blue concentration on decolourisation effectiveness. Tube test on Kimura medium was done in triplicate for five Evans blue concentrations (0.01, 0.025, 0.05, 0.075 and 0.1 g l^−1^). Bacterial strain inoculum was prepared from 48 h slants (Nutrient Agar (Fluka Biochemika)) washed with physiological salt solution. Suspension density was estimated to be 5 in the McFarland scale. Filter-sterilized colourants were added to tubes containing 10 ml of 48-h-old cultures in stationary growth phase (∼15 × 10^8^ cfu ml^−1^). After 6 days of incubation in 26 °C, absorbance was measured spectrophotometrically on UV–Vis Hitachi U-1900. Maximal absorbance was noticed at 606 nm. Percentage dye removal was calculated according to Formula 1.

The main experiment was prepared as previous test, but inoculum (1 ml) was added to 300 ml flasks containing 100 mL of Kimura medium. On the basis of results of the previous test, dye concentration chosen for this part of experiment was 0.05 g l^−1^. Water solution of diazo dye Evans blue (Sigma-Aldrich) was filter-sterilized (Millipore cellulose filters ∅ 0.20 μm) and added to 48-h-old bacterial samples (stationary growth phase). Cultures were incubated in static, semi-static and shaken conditions in 26° C. Dead biomass (autoclaved for 20 min in 121 °C, 1.5 atm.) was used for estimation of biosorption.

Absorbance was measured after 1, 6, 24, 48, 72, 96 and 120 h (UV–Vis spectrophotometer Hitachi U1900). The wavelength for Evans blue (606 nm) was determined experimentally as the wave with maximal absorbance. Percentage dye removal was calculated the same as previously according to Formula (1).

### Toxicity Test

The zootoxicity was evaluated using *Daphnia magna* (OECD 202) and phytotoxicity using OECD *Lemna* sp. growth inhibition test no. 221. Tests were performed in quadruple. EC_50_ value was estimated. On the basis of these data, acute toxicity unit (TU_a_) was calculated (Formula 2) and toxicity class was established.

2 $$ {\text{T}}{{\text{U}}_{\text{a}}} = {{{100}} \left/ {{{\text{E}}{{\text{C}}_{{50}}}}} \right.} $$

Where:

EC_50_: The effective concentration of a wastewater sample that causes an inhibition of test organism by 50 %.

Samples were classified according to ACE 89/BE 2/D3 Final Report Commission EC (TU_a_ < 0.4—non toxic (I class); 0.4 ≤ TU_a_ < 1.0—low toxicity (II class); 1.0 ≤ TU_a_ < 10—toxic (III class); 10 ≤ TU_a_ ≤ 100—high toxicity (IV class); TU_a_ > 100—extremely toxic (V class)).

## Results and Discussion

Common usage of dyes can cause environmental pollution, which needs effective cleanup technologies. Microbial techniques are a key research in the environmental sciences. Significant attention has been paid on dyes removal (biodegradation and decolourization) by living biomass of bacteria. Few researches have focused on dyes adsorption on dead bacterial cells. Pure strains as well as mixed cultures are studied and recommended. Most studied strains belong to genera: *Pseudomonas*, *Bacillus*, *Sphingomonas*, *Aeromonas*, *Citrobacter*, *Escherichia* and *Desulphovibrio*. In the present study, two *Pseudomonas* strains were tested. They are Gram-negative bacteria. As it is well known, Gram-negative bacteria have thin cell wall containing up to 10 % of peptidoglycan and outer membrane composed of proteins, phospholipids and lipopolysaccharides (Salyers and Whitt 2000; Schlegel 1993).

Bacterial strains were isolated from different scale wastewater treatment plants with different sewage loads. Both *Pseudomonas* isolates differ significantly in their decolourization potential. Fluorone dyes (erythrosine and Bengal rose) were almost two times better removed by strain SDz3 (Table 1) isolated from large-scale wastewater treatment plant in Katowice (80 % and more regardless of dyes concentration). For both strains, increase of erythrosine concentration caused decrease of decolourization and at the same time, Bengal rose was more effectively removed in samples with higher concentration.

**Table 1:** Removal of different concentrations of dyes in tube after 6 days test

Studied dyes	Strain Sz6		Strain SDz3	
	Initial dye concentration			
	0.05 g l^−1^	0.1 g l^−1^	0.05 g l^−1^	0.1 g l^−1^
	Dye removal [%]			
Erythrosine	52.02 ± 1.47	26.06 ± 0.98	92.66 ± 0.81	82.39 ± 1.03
Bengal rose	40.08 ± 1.96	55.25 ± 1.04	78.02 ± 1.11	87.21 ± 0.56
Crystal violet	98.31 ± 0.63	99.01 ± 0.33	89.00 ± 0.49	62.33 ± 0.96
Brillant green	97.55 ± 0.36	96. 47 ± 0.57	93.07 ± 0.23	46.05 ± 0.63
Evans blue	98.27 ± 0.71	84.01 ± 0.21	72.52 ± 0.74	48.08 ± 0.22

Triphenylmethane dyes as brilliant green and crystal violet were also tested. Brilliant green has two ethyl groups connected with two phenyl rings when crystal violet has three methyl groups connected with all three phenyl rings. Strain Sz6 removed almost all colour (more than 96 %) in samples with triphenylmethane dyes regardless of dye concentration and structure. Similar results were observed for SDz3 but only in samples with dyes concentration 0.05 g l^−1^. Higher content of dyes (0.1 g l^−1^) was removed in less than 63 % (62 % of crystal violet and 46 % of brilliant green). There are many results corroborating that dye structure and presence of different functional groups have influence on decolourization process (Sani and Banerjee 1999; An et al. 2002; Jang et al. 2005; Sharma et al. 2004; Tychanowicz et al. 2004). Bacterial strain *Citrobacter* sp. studied by An et al. (2002) removed faster and more effectively structurally simpler crystal violet and methyl red than more complicated gentian violet, malachite green and brilliant green. Connection of dye chemical structure and rate of decolourization was reported also by Sani and Banerjee (1999) for *Kurthia* sp. In samples with dyes concentration of 10 μM (≈5 mg l^−1^) after 30 min of incubation colour removal ranged from 8 % for structurally complicated ethyl violet to 100 % for structurally simpler brilliant green.

The same as for triphenylmethane dyes diazo Evans blue was better removed by strain Sz6 isolated from small wastewater treatment plants purifying mainly domestic wastewater. Triphenylmethane and azo dyes commonly used in textile colouring processes are released during cloth washing and that is why colourant concentration in domestic wastewater can be high. Strain Sz6 seems to be better adapted to higher concentration of triphenylmethane brilliant green and azo Evans blue than SDz3.

**Fig. 1:**
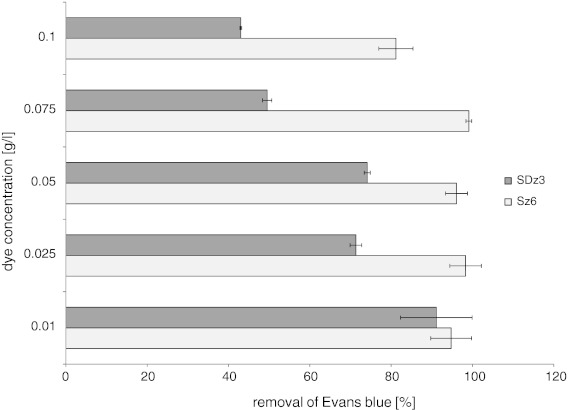
Influence of Evans blue concentration on *Pseudomonas* decolourization effectiveness

**Fig. 2:**
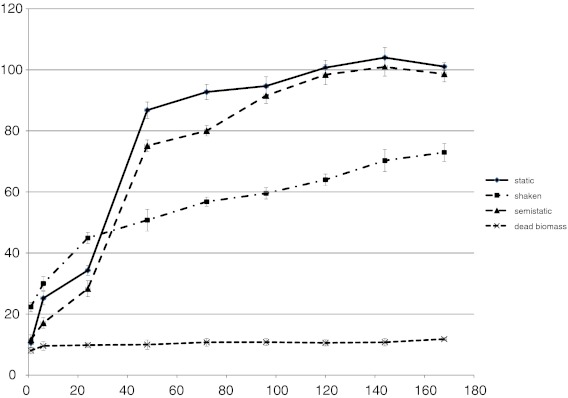
Decolourization of Evans blue by strain SDz3

**Fig. 3:**
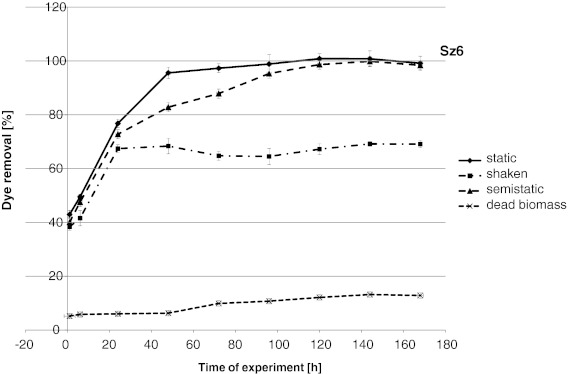
Decolourization of Evans blue by strain Sz6

Removal of increasing concentrations of Evans blue by Sz6 and SDz3 strains was also examined (Fig. 1). As in the previous test, all used concentrations were better removed by strain Sz6. Evans blue decolourization effectiveness by this strain reached 94–99 % in concentrations 0.01–0.075 g l^−1^ and 81 % in samples with 0.1 g l^−1^. Strain SDz3 removed more than 90 % of tested dye only in sample with 0.01 g l^−1^ of Evans blue. In other samples, it was less than 75 % (in samples with 0.075 and 0.1 g l^−1^ it was only 49 and 43 % respectively). On the basis of these results and preliminary test, the main experiment was done with 0.05 g l^−1^ of Evans blue. Biodegradation and biotransformation may be anaerobic, aerobic or a combination of these two. The main experiment was concentrated on Evans blue decolourization in different process conditions. Under anaerobic conditions, many bacteria reduce azo dyes by unspecific cytoplasmic azoreductases. Generally, increase of oxygen concentration decrease colour removal by live bacterial cells, because of direct inhibition of the azoreductase or preferential reduction of oxygen rather than the azo derivatives (Srinivasan and Viraraghavan 2010). Both tested strains removed all added dye in static and semi-static conditions after 120 h (Figs. 2 and 3). As it was shown previously, Sz6 was better adapted to the presence of Evans blue and removed the dye more effectively at the beginning of experiment (Fig. 3). In static and semi-static conditions up to 24 h, removal of Evans blue by Sz6 was more than two times higher than that reached in sample with SDz3 (34.3 and 76.7 % in static conditions and 28.3 and 72.7 % in semi-static conditions, respectively). After 48 h, strain SDz3 removed 86.8 % in static and 75.1 % in semi-static conditions (Fig. 2). At the same time, Sz6 removed 95.6 and 82.8 % of Evans blue, respectively. It was observed by Knapp et al. (1995) that the decolourization of azo dyes occurs on anaerobic as well as under semi-aerobic conditions.

Decolourization effectiveness in shaken samples (Figs. 2 and 3) up to 72 h of experiment was more than 10 % higher for strain Sz6 than for SDz3. About 70 % of Evans blue was removed by both strains (decolourization reached 69.1 % for Sz6 and 72.9 % for SDz3) and final decolourization effectiveness in shaken samples was more than 30 % lower than in other samples with living biomass. Aerobic *Pseudomonas* strains were more effective in static and semi-static conditions. The consequence of samples agitation is improving their oxygenation, which can also reduce azoreductase activity. Biological degradation of azo dyes is well documented as a reductive process. Moosvi et al. (2005) and Nigam et al. (1996) reported that although many of the cultures were able to grow aerobically, decolourization was achieved only under anaerobic conditions (Saratale et al. 2011). Aerobic *Bacillus*, *Pseudomonas*, *Proteus* and *Aeromononas* were found to be effective in the anaerobic degradation of a number of azo dyes (Saratale et al. 2011). The present study confirms that regardless of the aerobic character of the used strain, process conditions favouring azoreductase activity are more important for effective removal. It was reported that (Saratale et al. 2011) under aerobic conditions mono- and dioxygenase enzymes catalyse the incorporation of oxygen from O_2_ into the aromatic ring of dyes. Some aerobic bacteria are able to reduce azo compounds with the help of oxygen-catalysed azoreductase and produce aromatic amines. Aerobic azoreductase is able to use both NADPH and NADH as cofactors and reductively cleave carboxylated and sulphonated analogues of growth compounds (Saratale et al. 2011). This type of azoreductase activity was well reported for some *Pseudomonas* species (Stolz 2001; Saratale et al. 2011). These bacteria cannot use azo dyes as a growth substrate (Stolz 2001). McMullan et al. (2001) reported that there are a few *Pseudomonas* bacteria that use azo dyes as a sole carbon source and cleave N═N bonds reductively and utilise amines as a source of carbon and energy for their growth (Saratale et al. 2011).

Regardless of dye removal effectiveness, it is necessary to evaluate toxicity of process end products. Many bacteria strains reduce azo dyes to toxic, mutagenic and carcinogenic colourless aromatic amines (Srinivasan and Viraraghavan 2010). These intermediate metabolites can be further degraded mainly aerobically or rarely anaerobically (Saratale et al. 2011). Finally, effectiveness of decolourization of both studied *Pseudomonas* strains was comparable but differences in zootoxicity were observed (Table 2). Increase of zootoxicity from IV to V class was observed in static samples with strain Sz6 when in static samples with strain SDz3 decrease to III class was noticed. In semi-static and shaken samples with Sz6, no changes of zootoxicity were observed but samples with SDz3 were again classified to III class. Results of phytotoxicity test were comparable for both strains (decrease to I class in static and semi-static samples, and no changes in toxicity of shaken samples were observed).

**Table 2:** Results of toxicity tests

Strain	Culture conditions	*Daphnia magna*			*Lemna minor*		
		EC_50_	TU_a_	Toxicity class	EC_50_	TU_a_	Toxicity class
Sz6	Static	0.8 ± 0.32	125	V	n.d.^a^	<0.4	I
	Semi-static	9.43 ± 1.08	10.6	IV	n.d.^a^	<0.4	I
	Shaken	4.69 ± 1.70	21.3	IV	20.00 ± 4.10	5	III
	Dead biomass	11.36 ± 0.96	8.8	III	23.26 ± 8.20	4.3	III
SDz3	Static	12.5 ± 2.50	8.0	III	n.d.^a^	<0.4	I
	Semi-static	37.04 ± 2.80	2.7	III	n.d.^a^	<0.4	I
	Shaken	18.87 ± 3.20	5.3	III	76.92 ± 8.30	1.3	III
	Dead biomass	10.64 ± 1.80	9.4	III	24.39 ± 9.50	4.1	III
	Control with Evans blue	9.43 ± 0.22	10.6	IV	22.22 ± 2.10	4.5	III

^a^Not detected

Dead biomass is commonly used for evaluation of pollutants adsorption. As is described in the literature (Srinivasan and Viraraghavan 2010) dye uptake by dead (autoclaved) cells is extracellular. Cell wall structure play main role in biosorption on dead biomass.

In the present study, samples with autoclaved biomass removed only up to 13 % of used dye regardless of used strain (Figs. 2 and 3). It is necessary to remember that thermal processes change physicochemical properties of biological material that is connected with changes of sorption properties. Gram-negative bacteria (e.g., *Pseudomonas* sp.) have a higher adsorption capacity than Gram-positive bacteria due to higher lipid contents in the cell wall (Srinivasan and Viraraghavan 2010). Evans blue as anionic dye can be also bound by positively charged amine groups. Chemical functional groups of the bacterial cell wall include amine as well as carboxyl, phosphonate, sulphonate and hydroxyl groups which are responsible for dye binding (Srinivasan and Viraraghavan 2010).

Sorption of dyes on dead biomass was low (up to 13 %) but changes in zootoxicity (Table 2) were observed (decrease to III class). Phytotoxicity of samples was the same as in the control (Table 2).

## Conclusions

The present study confirms high effectiveness of *Pseudomonas* strains in dyes removal. The main experiment was focused on decolourization of azo Evans blue in different aeration conditions. In spite of the aerobic character of studied *Pseudomonas* strains, the best results were reached in static and semi-static conditions, confirming azoreductase contribution in the decolourization process. Both tested strains removed more than 85 % of used dye after 48 h and all colour after 120 h. Besides process effectiveness, ecotoxicological impact of cleaned wastewater on surface water deposits is essential. Strain Sz6 was very effective but zootoxicity test with *D. magna* classified cleaned samples to V class of toxicity in comparison with control classified to IV class. Decrease of zootoxicity was noticed for highly effective strain SDz3. Optimization of process conditions for the most promising strain SDz3 should be thoroughly investigated.

## References

[CR1] An SY, Min SK, Cha IH, Choi YL, Cho YS, Kim CH, Lee YC (2002). Decolorization of triphenylmethane and azo dyes by *Citrobacter* sp. Biotechnology Letters.

[CR2] Bin Y, Jiti Z, Jing W, Cuihong D, Hongman H, Zhiyong S, Yongming B (2004). Expression and characteristics of the gene encoding azoreductase from *Rhodobacter sphaeroides*. Microbiology Letters.

[CR3] Eichlerova I, Homolka L, Nerud F (2006). Synthetic dye decolorization capacity of white rot fungus *Dichomitus squalens*. Bioresource Technology.

[CR4] Fu Y, Viraraghavan T (2001). Fungal decolorization of dye wastewater: a review. Bioresource Technology.

[CR5] Gill PK, Arora DS, Chander M (2001). Biodecolourization of azo and triphenylmethane dyes by *Dichomitus squalens* and *Phelbia* spp. Journal of Industrial Microbiology & Biotechnology.

[CR6] Hu TL (2001). Kinetics of azoreductase and assessment of toxicity of metabolic products from azo dyes by *Pseudomonas luteola*. Water Science and Technology.

[CR7] Jang, M. S., Lee, Y. M., Kim, Ch. H., Lee, J. H., Kang, D. W., Kim, S. J., et al. (2005). Triphenylmethane reductase from *Citrobacter* sp. Strain KCTC 18061P: purification, characterization, gene cloning, and overexpression of functional protein in *Escherichia coli*. *Applied and Environmental Microbiology* 7955–7960 10.1128/AEM.71.12.7955-7960.2005 PMC1317337 16332773

[CR8] Knapp JS, Newby PS, Reece LP (1995). Decolorization of wood-rotting basidomycete fungi. Enzyme and Microbial Technology.

[CR9] McMullan G, Meehan C, Conneely A, Kirby N, Robinson T, Nigam P, Banat IM, Marchant R, Smyth WF (2001). Microbial decolorisation and degradation of textile dyes. Applied Microbiology and Biotechnology.

[CR10] Moosvi S, Keharia H, Madamwar D (2005). Decolourization of textile dye reactive violet 5 by a newly isolated bacterial consortium RVM 11.1. World Journal of Microbiology and Biotechnology.

[CR11] Moreira MT, Mielgo I, Feijoo G, Lema JM (2000). Evaluation of different fungal strains in the decolourization of synthetic dyes. Biotechnology Letters.

[CR12] Nigam P, McMullan G, Banat IM, Marchant R (1996). Decolorization of effluent from the textile industry by a microbial consortium. Biotechnology Letters.

[CR13] Padamavathy S, Sandhya S, Swaminathan K, Subrahmanyam YV, Kaul SN (2003). Comparison of decolorization of reactive azo dyes by microorganisms isolated from various source. Journal of Environmental Sciences.

[CR14] Pointing SB, Vrijmoed LLP (2000). Decolorization of azo and triphenylmethane dyes by *Pycnoporus sanguineus* producing laccase as the sole phenoloxidase. World Journal of Microbiology and Biotechnology.

[CR15] Salyers, A. A., Whitt, D. D. (2000). Microbiology: diversity, disease, and the environment. 1st edition. Wiley

[CR16] Sani RK, Banerjee UC (1999). Decolorization of triphenylmethane dyes and textile and dye-stuff effluent by *Kurthia* sp. Enzyme and Microbial Technology.

[CR17] Saratale RG, Saratale GD, Chang JS, Govindwar SP (2011). Bacterial decolourization and degradation of azo dyes: a review. Journal of the Taiwan Institute of Chemical Engineers.

[CR18] Schlegel, H. G. (1993). *General microbiology*, 7th edition. Cambridge University Press

[CR19] Sharma DK, Saini HS, Singh M, Chimni SS, Chandha BS (2004). Isolation and characterization of microorganisms capable of decolorizing various triphenylmethane dyes. Journal of Basic Microbiology.

[CR20] Somasiri W, Ruan W, Xiufen L, Jian C (2006). Decolourization of textile wastewater containing acid dyes in UASB reactor system under mixed anaerobic granular sludge. Electronic Journal of Environmental, Agricultural and Food Chemistry.

[CR21] Srinivasan A, Viraraghavan T (2010). Decolorization of dye wastewater by biosorbents: a review. Journal of Environmental Management.

[CR22] Stolz A (2001). Basic and applied aspects in the microbial degradation of azo dyes. Applied Microbiology and Biotechnology.

[CR23] Swamy J, Ramsay JA (1999). The evaluation of white-rot fungi in the decoloration of textile dyes. Enzyme and Microbial Technology.

[CR24] Tychanowicz GK, Zilly A, Marquez de Souza CG, Peralta RM (2004). Decolorization of industrial dyes by solid-state cultures of *Pleurotus pulmonaris*. Process Biochemistry.

[CR25] Van der Zee FP, Villaverde S (2005). Combined anaerobic–aerobic treatment of azo dyes—a short review of bioreactor studies. Water Research.

[CR26] Verma P, Madamwar D (2003). Decolorization of synthetic dyes by a newly isolated strain of *Serratia maerascens*. World Journal of Microbiology and Biotechnology.

